# Determinants of Laypersons’ Trust in Medical Decision Aids: Randomized Controlled Trial

**DOI:** 10.2196/35219

**Published:** 2022-05-03

**Authors:** Marvin Kopka, Malte L Schmieding, Tobias Rieger, Eileen Roesler, Felix Balzer, Markus A Feufel

**Affiliations:** 1 Institute of Medical Informatics Charité – Universitätsmedizin Berlin, corporate member of Freie Universität Berlin and Humboldt-Universität zu Berlin Berlin Germany; 2 Cognitive Psychology and Ergonomics Department of Psychology and Ergonomics (IPA) Technische Universität Berlin Berlin Germany; 3 Work, Engineering and Organizational Psychology Department of Psychology and Ergonomics (IPA) Technische Universität Berlin Berlin Germany; 4 Division of Ergonomics Department of Psychology and Ergonomics (IPA) Technische Universität Berlin Berlin Germany

**Keywords:** symptom checkers, disposition advice, anthropomorphism, artificial intelligence, urgency assessment, patient-centered care, human-computer interaction, consumer health, information technology, IT, mobile phone

## Abstract

**Background:**

Symptom checker apps are patient-facing decision support systems aimed at providing advice to laypersons on whether, where, and how to seek health care (disposition advice). Such advice can improve laypersons’ self-assessment and ultimately improve medical outcomes. Past research has mainly focused on the accuracy of symptom checker apps’ suggestions. To support decision-making, such apps need to provide not only accurate but also trustworthy advice. To date, only few studies have addressed the question of the extent to which laypersons trust symptom checker app advice or the factors that moderate their trust. Studies on general decision support systems have shown that framing automated systems (anthropomorphic or emphasizing expertise), for example, by using icons symbolizing artificial intelligence (AI), affects users’ trust.

**Objective:**

This study aims to identify the factors influencing laypersons’ trust in the advice provided by symptom checker apps. Primarily, we investigated whether designs using anthropomorphic framing or framing the app as an AI increases users’ trust compared with no such framing.

**Methods:**

Through a web-based survey, we recruited 494 US residents with no professional medical training. The participants had to first appraise the urgency of a fictitious patient description (case vignette). Subsequently, a decision aid (mock symptom checker app) provided disposition advice contradicting the participants’ appraisal, and they had to subsequently reappraise the vignette. Participants were randomized into 3 groups: 2 experimental groups using visual framing (anthropomorphic, 160/494, 32.4%, vs AI, 161/494, 32.6%) and a neutral group without such framing (173/494, 35%).

**Results:**

Most participants (384/494, 77.7%) followed the decision aid’s advice, regardless of its urgency level. Neither anthropomorphic framing (odds ratio 1.120, 95% CI 0.664-1.897) nor framing as AI (odds ratio 0.942, 95% CI 0.565-1.570) increased behavioral or subjective trust (*P*=.99) compared with the no-frame condition. Even participants who were extremely certain in their own decisions (ie, 100% certain) commonly changed it in favor of the symptom checker’s advice (19/34, 56%). Propensity to trust and eHealth literacy were associated with increased subjective trust in the symptom checker (propensity to trust *b*=0.25; eHealth literacy *b*=0.2), whereas sociodemographic variables showed no such link with either subjective or behavioral trust.

**Conclusions:**

Contrary to our expectation, neither the anthropomorphic framing nor the emphasis on AI increased trust in symptom checker advice compared with that of a neutral control condition. However, independent of the interface, most participants trusted the mock app’s advice, even when they were very certain of their own assessment. Thus, the question arises as to whether laypersons use such symptom checkers as substitutes rather than as aids in their own decision-making. With trust in symptom checkers already high at baseline, the benefit of symptom checkers depends on interface designs that enable users to adequately calibrate their trust levels during usage.

**Trial Registration:**

Deutsches Register Klinischer Studien DRKS00028561; https://tinyurl.com/rv4utcfb (retrospectively registered).

## Introduction

### Background

Patients are increasingly searching for health information on the web before seeking medical care [1-3]. As an alternative to commercial search engines, patient-facing decision support systems called symptom checkers were developed to provide the first access point to health-related information. These tools are targeted at laypersons and ask users to enter their signs and symptoms before presenting preliminary diagnoses and an assessment of the level of care to seek [4]. The latter assessment, the so-called disposition or urgency advice, is arguably the more important function of symptom checkers, as it could prevent unnecessary visits and direct patients toward the appropriate health care facility, thus reducing the burden on the health care system [5,6].

### Related Work

Symptom checkers have mostly been investigated in terms of accuracy; user characteristics; and, occasionally, their effect on user care-seeking behavior. We will report on these findings in turn. In non–industry-funded studies, their *accuracy* appears to be mediocre: Semigran et al [6] found that disposition advice of apps is accurate at 57% on average, Yu et al [7] identified an accuracy between 50% and 74% for emergency cases, and Hill et al [8] found appropriate disposition advice to be provided in 49% of case evaluations on average. Although the symptom checker accuracy in these studies is mediocre, the range is very broad, and some symptom checkers perform well. For example, Ceney et al [9] found a disposition accuracy of up to 90% for a system that performs best in urgency assessment. At the health system level, evidence is still inconclusive whether symptom checkers bear the potential to make patient journeys more efficient and decrease the burden on health care services, with a study on telephone triage suggesting a redistribution rather than a reduction in health care workload [4,10-12]. Given that the current reliability of symptom checkers seems rather low on average, 2 (systematic) reviews advise against using these tools in lieu of current assessment models [13,14].

Concerning user characteristics, research has found that symptom checker *users* are predominantly female, more often young than old, and more often have a higher than a lower level of education [15,16]. In terms of *behavioral effects*, one study showed that most users plan to follow the received advice [17]. Another study by Winn et al [18] found that the perceived urgency of symptoms decreased after using a symptom checker. However, the advice given by the symptom checker was not recorded in that study, and it remains unclear whether users are more prone to lower urgency advice or whether they might have overestimated the urgency in their initial assessment. A vignette-based experimental study found that on average, symptom checkers currently do not outperform laypersons in terms of disposition accuracy. However, best-in-class apps seem superior to laypersons [19,20].

In addition to a system’s accuracy, which is well known to affect behavior, subjective trust (ie, self-reported trust in automated systems; see the study by Schaefer et al [21]) is a key factor determining whether humans follow advice from decision aids or rely on automated systems (behavioral trust). Trust in automation has been shown to be influenced by several factors, which can be divided into performance based (eg, reliability) and attribute based (eg, appearance) [21]. Although symptom checker research has so far focused on performance-based factors, studies on the influence of attribute-based factors are mostly missing. In general automation research, anthropomorphism—making the automation appear human like—has been identified as one of many potential influences [22]. There are several methods for designing human-like systems or framing them as such; however, visual anthropomorphism is the easiest to include in a symptom checker (eg, using a picture of a person on the user interface). The direction of the relationship between visual anthropomorphism and trust seems to vary. In a study by de Visser et al [23], trust was lower for anthropomorphic interfaces compared with technical systems. However, this is only the case when the system’s reliability is high. With decreasing reliability, trust decreased less steeply for the anthropomorphic system than for the technical system, suggesting a resilient influence of anthropomorphism, which could be replicated in another study [24].

In contrast, in a medical decision-making task, Pak et al [25] found that trust and follow rates, with constant reliability of 67%, were higher when the decision support system’s interface included the image of a physician. These contradictory findings might be explained by Hertz and Wiese [26], who found that people preferred assistive agents that were thought to have the greatest expertise for a specific task. For medical decision-making, health care professionals are highly trusted, and patients seem to ascribe greater expertise to physicians than to self-assessment apps, whereas in other use cases, such as analytical and computational tasks, users might find assistance from a nonhuman agent more trustworthy [1,27]. In terms of symptom checkers, anthropomorphic framing could be used to increase expertise perception because of humanization (ie, making it more human like) or technological framing (eg, artificial intelligence [AI]) because of technologization (ie, emphasizing its technological nature so that it is seen as an expert system). Indeed, some symptom checkers, such as Symptomate [28], have already emphasized using AI algorithms, which are commonly used as a buzzword for machines imitating human intelligence [29]. Although transparent communication of using AI in applications will soon be required by law [30], the design of such systems often hints at displaying AI use to enhance trust because of increased expertise perception [31]. On the basis of these findings, will showing an image of a physician in symptom checkers make them more trustworthy? Could trust also be enhanced by emphasizing that symptom checkers base their recommendations on AI?

### Aim of This Study

This study aimed to examine the influence of framing effects on subjective trust in symptom checkers and the behavioral consequences of trust (ie, dependence and following behavior), which are strongly related [32]. Higher trust is particularly useful when using highly accurate symptom checkers, as patient outcomes can only be improved by following correct (and safe) advice. However, when a symptom checker does not perform well, high trust can also be dangerous in the case of incorrect advice (eg, recommending self-care while emergency care is required). Thus, our study aimed to identify potential factors influencing users’ trust in these decision aids apart from system accuracy.

As trust in physicians is generally higher than in computerized decision aids [27], we were particularly interested in assessing whether anthropomorphic framing (ie, displaying an image of a physician as a human expert decision maker on the user interface) leads to increased trust in decision aids. Furthermore, we examined whether framing the symptom checker as being based on AI increases users’ trust. We hypothesized that anthropomorphism would increase participants’ subjective trust in the app and the proportion of participants following the app’s advice (behavioral trust). We expected the same effect (higher subjective and behavioral trust) when framing the symptom checker as AI. As Winn et al [18] showed that users commonly decreased their appraised urgency level after symptom checker use, we explored whether users might be more prone to follow a symptom checker when its urgency appraisal is lower than their own. Kopka and colleagues [33] found that most laypersons are certain in their urgency assessment and that in absolute numbers, laypersons make most errors when they are certain of their appraisal. For this reason, we also examined whether users tended to accept advice from such decision aids when they were already certain of their own judgment. We expected that users’ inclination to follow a decision aid’s advice would decrease with higher decisional certainty, as users tend to rely on automation when they are not confident but solve tasks manually when they are confident [34,35]. Finally, we explored the association between demographical and other interindividual variables and trust.

## Methods

### Ethics Approval and Consent to Participate

This study was approved by the Ethics Committee of the Department of Psychology and Ergonomics (Institut für Psychologie und Arbeitswissenschaft [IPA]) at Technische Universität Berlin (tracking number: FEU_9_210315). Participants volunteered to participate in the survey, and informed consent was required. On the first page, participants were told about the investigator, the study’s purpose, what data were to be collected during the study, and where and for how long they would be stored. On the second page, participants were informed about the duration of the survey (approximately 5 minutes) and received additional information regarding the scope and use of attention checks.

### Participants

Yee et al [36] found that the effect size for showing a human in an interface on subjective trust was Cohen's *d*=0.28. On the basis of an a priori power analysis for independent *t* tests with an assumed Cronbach α of .05 and a power of 1−β=0.80, we aimed to sample at least 477 (n=159, n=159, and n=159 for the 3 groups, respectively) participants to detect differences between the 3 groups (2 experimental groups and 1 control group). We expected some participants to fail attention checks (items that were embedded in the survey questions and asked participants to select a particular option, eg, “Please select Disagree”); therefore, we oversampled by 10%. To avoid participants’ decisions being influenced by their residential country or ability to understand the scenario, only US residents fluent in English were eligible. They also had to participate in the web-based questionnaire on a desktop device or tablet as the survey’s graphical elements could not be reliably displayed on smartphone devices. Another requirement was not being a medical professional (ie, nurse, paramedic, and physician). We sampled participants using Prolific [37], a platform characterized by high data quality [38], starting on Saturday, May 15, 2021, at 5 PM Eastern Daylight Time and on Sunday, May 16, 2021, at 4 PM Eastern Daylight Time. We chose these days as Casey et al [39] have shown that the samples recruited via the web are more diverse during the weekend than on working days. Following Ho et al [40], participants were compensated £0.70 (US $0.91) for their participation and received an additional £0.18 (US $0.24) as an incentive for the correct decision (ie, selecting self-care in their last appraisal) to increase data quality through attentive participation.

### Design

**Figure 1:**
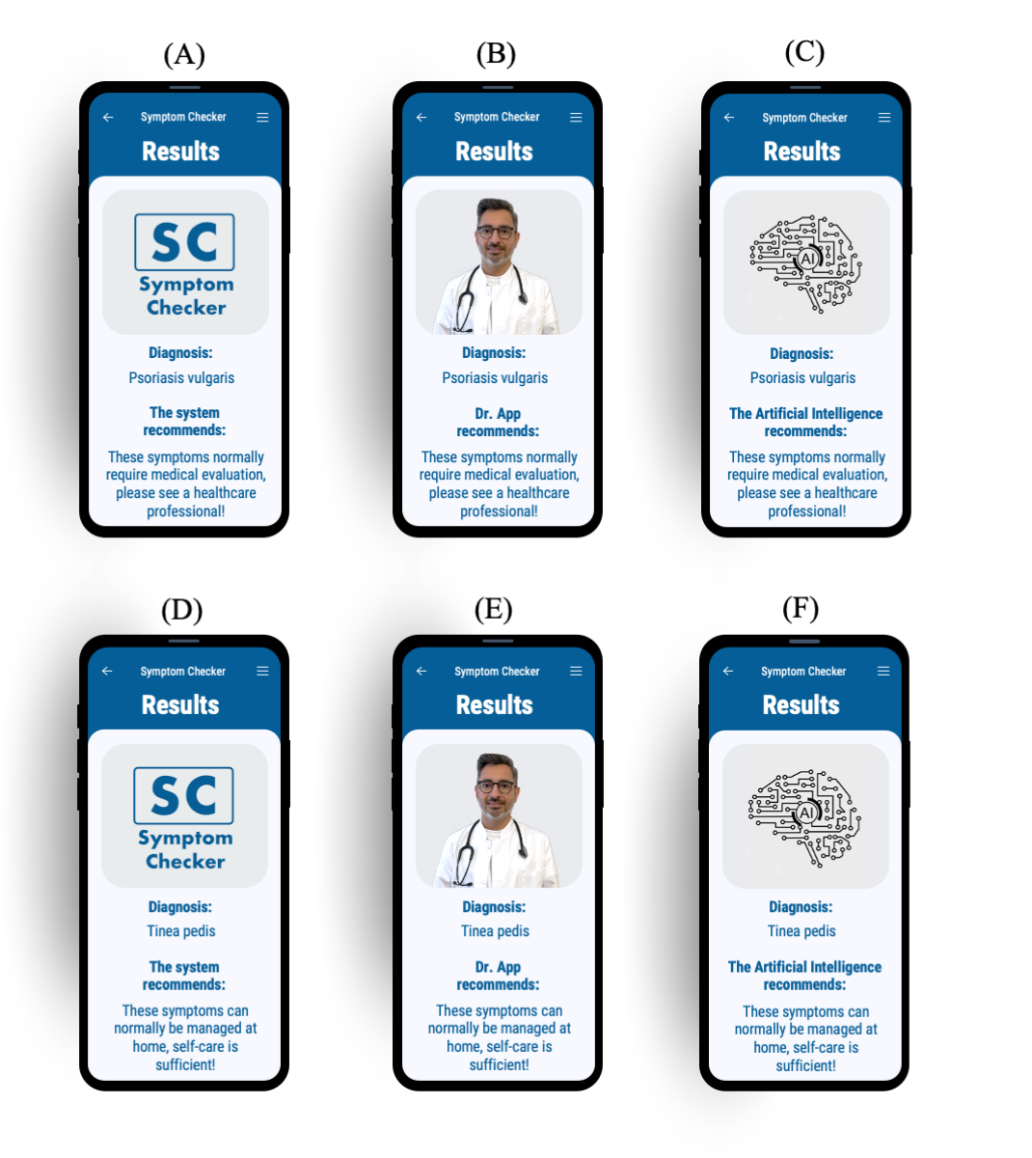
Interfaces: participants were asked about their initial appraisal and received contrary advice from the results screen of a mock symptom checker. Screens (A), (B), and (C) present advice to seek health care, whereas screens (D), (E), and (F) recommend that self-care is sufficient. Participants were randomized and received advice from a neutral (A) and (D), anthropomorphic (B) and (E), or artificial intelligence-framed (C) and (F) screen.

We used a 1-factorial experimental design with factor framing and factor levels of anthropomorphic framing and framing as AI along with a control group (allocation ratio 1:1:1). These were manipulated by integrating a picture of a physician, an iconographic representation of AI similar to that displayed by Symptomate [28], or a mock company logo into the mock symptom checker’s advice screen (Figure 1). Participants were automatically randomly assigned (simple randomization) to one of these levels using a randomization tool integrated into the Unipark Enterprise Feedback Suite (EFS) Survey. In every condition, they had to appraise one and the same case vignette by deciding whether the fictitious patient required health care or self-care was sufficient. Although there are other urgency levels in symptom checkers, we chose this binary decision as the question of whether to seek care at all is the first decision patients must make [15,20]. The participants were tasked to appraise the case vignette twice: once before receiving advice from the decision aid (initial stand-alone assessment) and once after receiving the advice. The decision aid’s advice was programmed to always contradict the participant’s stand-alone assessment. The dependent variables were subjective and behavioral trust (ie, whether the participant followed the advice of the decision aid).

### Materials

We chose one specific case vignette, originally used by Hill et al [8] to assess symptom checker accuracy, as it showed a high item variance; that is, participants in an unpublished pretest with 56 participants were about equally split in their appraisal of whether the fictitious patient required health care. Owing to the high item variance, this case vignette allowed us to capture the potential influence of the advice’s urgency (ie, higher or lower urgency) on users’ trust. The case vignette describes the typical presentation of fungal skin infection (tinea pedis), colloquially known as athlete’s foot: “A 33-year-old male has scaly skin between the toes. The skin is a little itchy and turns soft and white when wetted. The skin has an odour.” According to the gold standard solution for this case vignette assigned by an expert panel [8], proper self-care was sufficient for this case. Although not necessary, we would also consider it appropriate to seek professional health care for the condition, as a physician could educate the patient on proper self-care options. Hence, it is neither negligent in regarding self-care as sufficient nor is it overcautious to deem the health care required when appraising this vignette. As the decision aid always disagreed with the participants’ initial assessment, it either gave higher or lower urgency advice depending on the participant’s initial stand-alone assessment. As most symptom checkers complement their disposition recommendation with a diagnostic assessment, our decision aid named a possible diagnosis congruent with the corresponding urgency level: when providing disposition advice more acute than the participant (ie, the symptom checker recommended seeking health care), the symptom checker provided the (made-up) diagnosis of psoriasis vulgaris along with the text, “These symptoms normally require medical evaluation, please see a healthcare professional!” For lower urgency, it returned the (original) diagnostic suggestion of tinea pedis along with the text, “These symptoms can normally be managed at home, self-care is sufficient!” The wording is based on a screening of different symptom checkers and represents a symbiosis between the advice given by Symptomate [28] and Ada [41].

As a decision aid, we created a mock app with a simple result presentation screen using PowerPoint (Microsoft Corporation) [42], Affinity Photo (Serif Ltd) [43], and Vectornator (Linearity) [44]. Participants could not interact with the decision aid to input information as not everyone would have entered the symptoms in the same way, and thus, the decision path would have differed. As this interaction influences trust [45], we tried to eliminate any resulting bias by presenting a results screen only. This design was inspired by Pak et al [25], who assessed anthropomorphism in a decision support system for diabetics using the picture of a physician. We designed our interfaces to include the same diagnoses and disposition advice with a picture of a mock symptom checker logo, a physician, or an icon representing AI (Figure 1). To ensure that the decision aid was displayed in the same way for all participants and that the results were not biased by different presentations on different phones, we placed the interface directly in a mock phone. The simulated phone could then be viewed on a computer or tablet. For the anthropomorphic condition, we chose the depiction of a young male physician based on the findings of a study by Pak et al [46], who found the depictions of a male physician embedded in a decision aid less susceptible to fluctuations in perceptions of trust as a function of the decision aid’s reliability, and depictions of younger agents exhibited fewer age differences in perceived trust than older agents.

Framing manipulation corresponds to the actual visual framing in a widely used symptom checker [28]. Although framing can be manipulated to a greater extent (eg, by presenting videos and stories [23]), we decided to use a picture only to represent currently applied practice. Therefore, the extent of our manipulation is similar to that of other studies that assessed the effects of anthropomorphism on trust in decision aids [25,47].

### Survey

A web-based survey was developed using Unipark EFS Survey [48]. All collected data were saved on the platform, and only the authors of this study had access to the data. We evaluated the usability and technical functionality of the questionnaire and then conducted a pilot study in which test participants were asked to provide feedback on any display problems, unclear questions or statements, or other issues that might have occurred. After these were resolved, the questionnaire was rolled out as a voluntary, open survey that was only accessible via the Prolific recruitment platform (initial contact). We did not advertise the survey in any other way than presenting it on the platform. Participants were presented with 1 questionnaire on each page; hence, the items per page ranged from 1 to 13 on a sum of 19 pages. They could return using browser buttons and review their answers, which were checked for completeness using the built-in function of the Unipark EFS Survey. Although the symptom checker interfaces were adapted to the participant’s responses (see the *Design* section and the *Materials* section), we did not use adaptive questioning to reduce the number of questions.

Survey visitor numbers were assessed by assigning participants an ID when opening the questionnaire. Most participants accessing the survey completed it (completion rate: 572/607, 94.2%).

### Dependent Measures

Subjective trust in the symptom checker app (primary outcome) was measured by adapting the Trust in Automated Systems Survey [49], which uses a 7-point Likert scale with 12 items; as suggested by Gutzwiller et al [50], we randomized the order in which items were presented to avoid a positive bias. Behavioral trust (secondary outcome) was measured using an adapted TNO trust task [51], as previously reported by several authors [23,24,47,52]. First, the participants had to rate the appropriate urgency level on their own. Afterward, they were shown the symptom checker app’s contradicting recommendation and had to make a final decision. We measured whether they changed their decision in favor of the decision aid’s advice and coded behavioral trust at the individual level as Boolean (true or false). We then determined the proportion of participants following the advice (follow rates) as a measure of behavioral trust at the group level.

### Procedure

After participants gave consent to participate, we surveyed their age, gender, educational background, and prior medical training. Next, participants were asked about their propensity to trust using the Propensity to Trust in Technology Scale with 6 items on a 5-point Likert scale [53] and their eHealth literacy using the eHealth Literacy Scale with 8 items on a 5-point Likert scale [54].

Afterward, the 2 urgency levels (*health care* and *self-care*) between which the participants had to choose when appraising the fictitious case vignette were explained, and participants’ understanding of these definitions was assured with multiple-choice questions (3 rewordings of the urgency level definitions to which participants had to assign the correct urgency level). Next, they were shown the case vignette, and they appraised its urgency and rated their decisional certainty using a visual analogue scale with values from 0 (minimum certainty) to 100 (maximum certainty). They then saw the results screen of the mock decision aid advising the opposite of their assessment with 1 of the 3 different designs (Figure 1) and had to make a final decision on the urgency level and again state their decisional certainty.

Thereafter, they were presented with the Trust in Automated Systems Survey and had the opportunity to provide feedback or any other comments in an open text field. Finally, to ensure that our intervention was successful, participants were asked to specify which image was embedded in the decision aid presented to them previously (manipulation check).

### Data Analysis

We cleaned and analyzed the data using base R (version 4.0.5) [55], the *tidyverse* packages [56], and *aod* [57]. For inferential analysis of continuous outcomes, we used a 1-way between-subjects ANOVA. For binary outcomes, we used dummy-coded binomial logistic regression and tested the coefficients using Wald chi-square tests. To test demographic and interindividual influences, we used multiple linear regression and multiple binomial logistic regression with standardized coefficients for better comparability. The effect coding scheme and results can be found in Multimedia Appendices 1-6. We used an effect coding scheme to compare each factor level to the mean of all factor levels. Thus, deviations from the mean can be quantified and tested for significance instead of performing group comparisons with a single, consistent reference category (as in dummy coding). For example, the coefficients and *P* values of gender 1 (Multimedia Appendix 1) represent the differences and significance tests of women compared with the mean of other genders. Similarly, the metrics of education 1 (Multimedia Appendix 2) represent differences between participants with a bachelor’s degree compared with the mean of all other education levels. Finally, we conducted sensitivity power analyses using the R package *pwr* [58] to estimate the population effect size for selected results that appeared statistically nonsignificant.

## Results

### Participant Characteristics

The survey was completed in 6 minutes and 19 seconds (Median, IQR 4 minutes, 36 seconds to 8 minutes, 46 seconds). Of the 607 individuals accessing the survey, 35 (5.8%) did not finish the questionnaire, 14 (2.3%) were excluded as they were trained medical professionals, 27 (4.4%) were excluded as they took part on a mobile phone, and 37 (6.1%) failed at least one of the attention checks. Therefore, of the 607 individuals, the total sample size was 494 (81.4%). Distributions of age, gender, level of education, propensity to trust, and eHealth literacy overall and in each of the 3 groups are reported in Table 1.

**Table 1:** Participant characteristics (N=494).

Characteristics			Control group (n=173)		Anthropomorphic (n=160)		Artificial intelligence (n=161)		Total
Age (years), mean (SD)			34.5 (13.8)		32.1 (12.5)		31.6 (12.2)		32.8 (12.9)
**Gender, n (%)**									
	Female	81 (46.8)		78 (48.8)		77 (47.8)		236 (47.8)	
	Male	87 (50.3)		80 (50)		82 (50.9)		249 (50.4)	
	Other	5 (2.9)		2 (1.3)		2 (1.2)		9 (1.8)	
**Education, n (%)**									
	Less than high school	0 (0)		4 (2.5)		3 (1.9)		7 (1.4)	
	High school graduate	25 (14.5)		12 (7.5)		20 (12.4)		57 (11.5)	
	College or associate degree	48 (27.7)		50 (31.3)		63 (39.1)		161 (32.6)	
	Bachelor degree	66 (38.2)		66 (41.3)		52 (32.3)		184 (37.2)	
	Graduate degree or higher	34 (19.7)		28 (17.5)		23 (14.3)		85 (17.2)	
**Prior medical training, n (%)**									
	No training	141 (81.5)		135 (84.4)		136 (84.3)		412 (83.4)	
	Basic first aid	32 (18.5)		25 (15.6)		25 (15.5)		82 (16.6)	
Propensity to Trust score^a^, mean (SD)			4.1 (0.5)		4.1 (0.5)		4.0 (0.5)		4.1 (0.5)
eHEALS^b^ score^c^, mean (SD)			30.5 (4.91)		30.0 (5.27)		30.1 (5.61)		30.2 (5.25)
**Initial assessment of the case vignette, n (%)**									
	Health care	60 (34.7)		62 (38.8)		65 (40.4)		187 (37.9)	
	Self-care	113 (65.3)		98 (61.3)		96 (59.6)		307 (62.1)	
Completion time (minutes), median (IQR)			6:32 (4:32-8:53)		6:09 (4:39-8:17)		6:18 (4:38-9:16)		6:19 (4:36-8:46)

^a^Propensity to Trust refers to the Propensity to Trust in Technology Scale, and possible scores range from 1 (low) to 5 (high).

^b^eHEALS: eHealth Literacy Scale.

^c^Possible scores range from 8 (low) to 40 (high).

Almost all participants (480/494, 97.2%) correctly recollected the image embedded in the mock decision aid (manipulation check).

### Influence of Framing on Participants’ Trust

#### Subjective Trust

**Figure 2:**
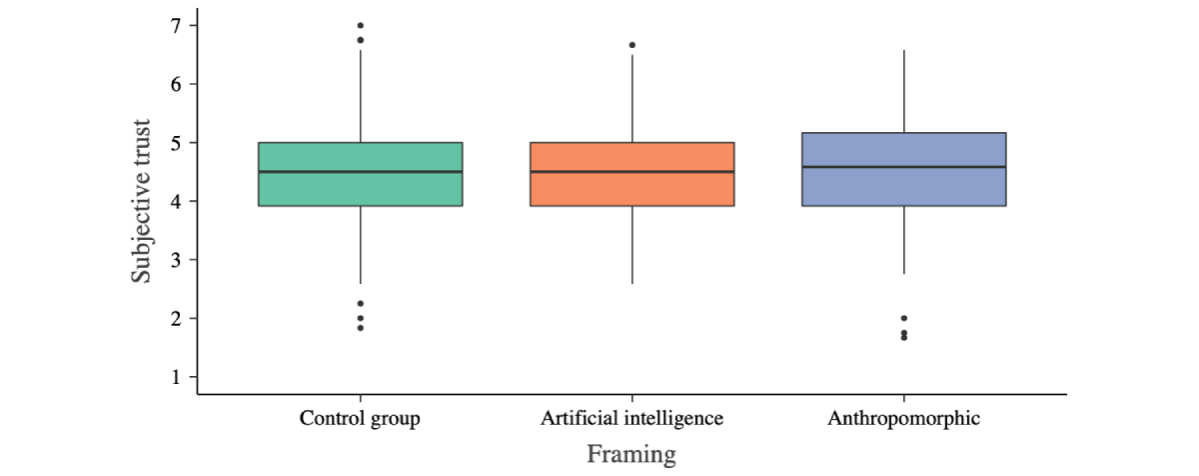
Subjective trust scores across the 3 study groups. Trust was operationalized using the Trust in Automated Systems Survey with a range from 1 (minimum trust) to 7 (maximum trust). The horizontal line in the box represents the median.

Descriptively, trust in all 3 framing conditions was very similar (anthropomorphic: mean 4.503, SD 0.922; AI: mean 4.495, SD 0.817; control: mean 4.508, SD 0.921; Figure 2). Framing had no significant effect on subjective trust (*F*_2,491_=0.009; *P*=.99; η^2^=0.00). On the basis of a sensitivity power analysis (α=.05; 1−β=0.80; anthropomorphic n=160, AI n=173, and control n=161), we estimated the effect size of possible differences between the groups to not be greater than η^2^=0.018.

#### Behavioral Trust

Most participants followed the decision aid’s advice and changed their urgency appraisal (384/494, 77.7%). Behavioral trust was slightly higher for the anthropomorphic system (127/160, 79.4%) than for the control group (134/173, 77.5%); however, the difference (odds ratio [OR] 1.120, 95% CI 0.664-1.897) was not statistically significant (*χ^2^*_1_=0.2; *P*=.67).

Behavioral trust was slightly lower for the AI system (123/161, 76.4%) than for the control group; however, the difference (OR 0.942, 95% CI 0.565-1.570) was not statistically significant (*χ^2^*_1_=0.053; *P*=.82), either.

### Influence of the Urgency Level Provided by the Decision Aid on Trust

We observed no differences in subjective trust between participants receiving advice of greater urgency (*health care required*) than their stand-alone initial assessment (*self-care sufficient*) (mean 4.5, SD 0.919) and those receiving less urgent advice (mean 4.5, SD 0.869). Concerning behavioral trust, the proportion of participants who followed more urgent advice was slightly lower (235/307, 76.5%) than the proportion who followed advice of lower urgency than their own initial stand-alone assessment (149/187, 79.7%).

### Influence of Participants’ Decisional Certainty on Trust

The participants of all 3 groups were certain about their initial stand-alone assessment (median 70, IQR 60-81). Only 12.8% (63/494) were unsure (ie, indicating a certainty of <50% about their appraisal). No differences in patterns were observed between the framing conditions.

**Figure 3:**
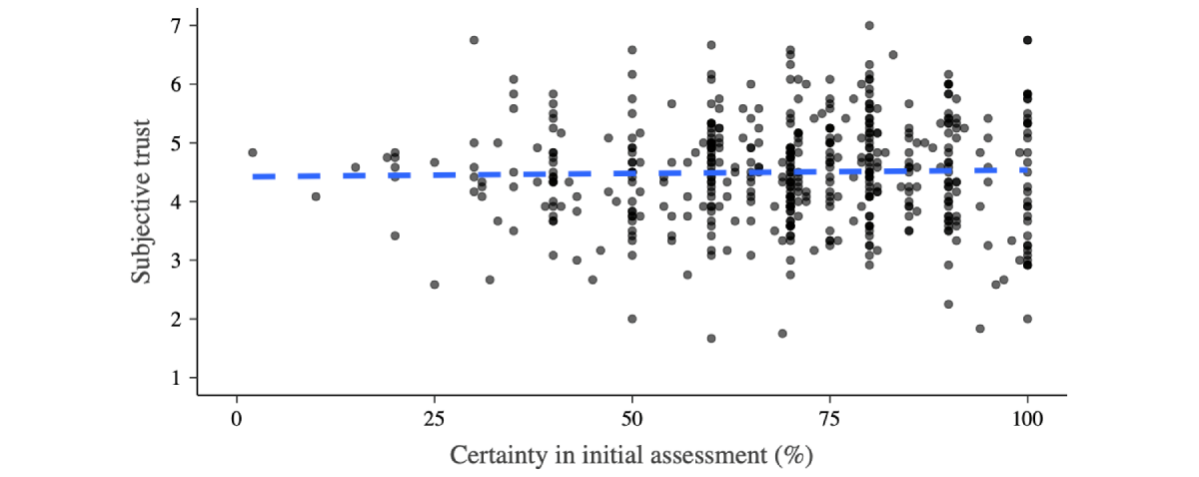
Subjective trust and participants’ certainty. Trust was operationalized using the Trust in Automated Systems Survey (range: 1-7). The dashed blue indicates a linear model for the association between participants’ certainty in their initial stand-alone appraisal of the case vignette and the subjective trust toward the decision aid.

**Figure 4:**
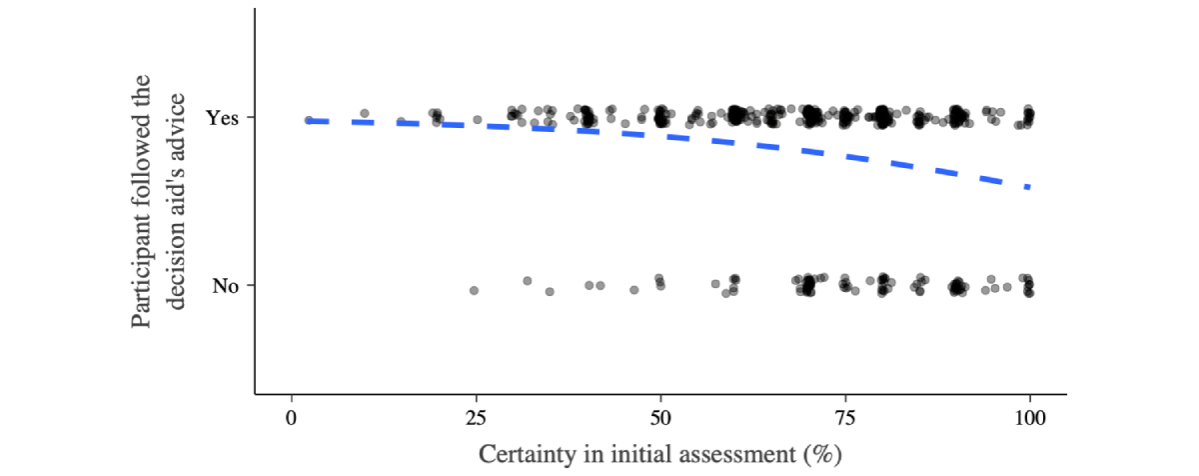
Behavioral trust and participants’ certainty. The dashed blue indicates a binomial logistic model for the association between participants’ certainty in their initial stand-alone appraisal of the case vignette and the behavioral trust toward the decision aid.

Participants’ certainty in their initial assessment was not associated with subjective trust in the decision aids (*R*^2^=0.001; Figure 3). With increasing decisional certainty, behavioral trust decreased (OR 0.966, 95% CI 0.952-0.979; *χ^2^*_1_=25.0; *P*<.001; McFadden *R*^2^=0.055). However, behavioral trust was high and remained >50% (19/34, 56%), even for participants indicating maximum decision certainty (100/100; Figure 4). There were no differences between the framing conditions.

### Demographic and Interindividual Influences on Trust

Neither demographic variables (age, gender, and education) nor basic first aid training was associated with subjective and behavioral trust in the symptom checker when controlling for the other variables. However, an individual’s propensity for trust and eHealth literacy increased subjective trust and were statistically significant (*P*<.001). However, these 2 variables did not have a statistically significant influence on behavioral trust (Multimedia Appendices 4 and 6).

## Discussion

### Principal Findings

#### Effect of Anthropomorphic or AI Framing

The aim of this study was to explore the factors influencing laypersons’ subjective and behavioral trust in symptom checkers. In particular, we examined the hypothesis that the common features of symptom checker interfaces that frame the system as either AI-based or anthropomorphic affect users’ trust in these systems. Our analysis does not support this hypothesis: we could not observe a difference in trust—neither subjective nor behavioral—between a neutral symptom checker interface (showing a mock company logo) and interfaces framed as either anthropomorphic or as using AI. This is in contrast to previous findings from other domains where anthropomorphism led to an increase or decrease in trust [23-25]. In addition, we expected that designing anthropomorphic decision aids in a clinical context would yield higher trust as symptom checker users trust physicians more than self-assessment apps [15,26,27,59]. However, our participants’ trust was unaffected by how the symptom checker was framed, indicating that users seem to perceive a symptom checker mainly as an app, regardless of whether a depiction of a physician or an AI icon is included. We did not find an effect of framing on subjective and behavioral trust in our study; however, we cannot rule out that framing might influence other variables in the trust formation process. For example, anthropomorphism has been shown to moderate the relationship between reliability and trust [23,24]: framing might moderate the impact of a decision aid’s reliability on trust; however, it might not be sufficient to build trust (in medical advice) on its own. Instead, other factors, such as explanations of the reasoning underlying the symptom checker’s advice, might help build trust more effectively [60].

#### Persuasive Power of Symptom Checker Apps

We found that most participants (384/494, 77.7%) followed the decision aid’s advice. This is in line with Verzantvoort et al [17], who reported a high intention of users to follow dispositional advice from a decision aid (65%). However, both findings stand in contrast to those indicating a low behavioral trust in symptom checkers; for example, Meyer et al [15] reported that only a minority of those advised by a symptom checker to visit the emergency department followed this advice, and Miller et al [61] found that most patients presenting to a primary care clinic stick to their stand-alone assessment when using a symptom checker in a primary care clinic’s waiting room. Taken together, these findings hint at symptom checker users’ behavioral trust being a function of the exact urgency decision and context of use: when users are undecided between seeking emergency or nonemergency care, they might depend less on the symptom checker’s advice compared with when choosing whether professional medical care is required at all or self-care is appropriate. Users might also be more inclined to accept guiding advice from symptom checker apps before arriving at a health care facility. Interestingly, a web search seems to change only few people's urgency level [62]; this indicates a difference in advice-taking between symptom checker use and general web search.

Another influencing factor on behavioral trust is decisional certainty; that is, whether users follow a symptom checker’s advice depends on how certain they are of their own stand-alone assessment. However, our findings hint at the high persuasive power of symptom checkers: although participants indicating maximum certainty in their own stand-alone assessment followed the advice less often than those indicating lower levels of certainty, most still changed their decision according to the decision aid’s recommendation. This finding is central as it emphasizes the impact symptom checkers may have on the urgency decision. Symptom checkers could not only assist when patients are uncertain whether and where to seek health care but also convince those who (wrongly) are very certain in their appraisal. This might prove very useful, as Kopka and colleagues [33] report that laypersons’ urgency errors are most frequent when they indicate high confidence in their stand-alone appraisal. In contrast, high dependence on symptom checkers potentially signifies laypersons using them as a replacement for decision-making rather than as a decision aid. This should be further investigated through research on the cognitive and metacognitive mechanisms with which laypersons monitor their own reasoning when confronted with advice from symptom checkers, similar to the Jussupow et al [63] study on an AI-based decision aid supporting physicians in diagnostic decisions. In addition, laypersons’ high dependence on symptom checkers emphasizes the need for a framework to identify and label those apps defying the general trend by proving them to be both accurate and safe to use, as currently, symptom checkers’ accuracy is being reported as mediocre in general, with only a few performing well [6-9,14].

#### Interindividual Variables' Effect on Trust in Symptom Checkers

A previous study indicated gender differences in appraising medical situations (eg, Cooper and Humphrey [64] showed that female participants assessed their urgency as more risk averse); however, we could not replicate this finding for trust. Our findings suggest that demographic and interindividual differences might be negligible when drafting recommendations on whether and how symptom checker apps should be designed. Although users who are generally more inclined to trust show higher subjective trust in symptom checkers, they do not seem to follow their advice more often, which might have methodological reasons; that is, the item terminology referencing technology too broadly [53,65]. Concerning the influence of eHealth literacy on trust, we observed that it increased subjective trust but not behavioral trust. Users with higher eHealth literacy might have more knowledge about eHealth applications and thus be more open to receiving advice from a decision aid while at the same time being more able to integrate a decision aid’s advice into their own decision-making rather than uncritically adopting the presented advice.

### Limitations

First, the intervention might not have been effective in producing meaningful differences. However, nearly all participants (480/494, 97.2%) were able to recall the picture they were presented with as part of the decision aid, thus proving that they took note of the depictions used for framing. Moreover, the results remained consistent, even if participants who could not recall the presented picture were excluded from the analysis. The framing itself represents another limitation. Although we followed the current practice and manipulation extent of previous studies, other interface and framing aspects are conceivable that may not have been captured in this study. For example, it would be interesting to assess whether personalized images (eg, patients’ own physicians) could increase their trust.

In our study, participants did not interact with the decision aid as we only presented a symptom checker’s results screen instead of letting them enter the data or symptoms into an actual app. This was done to keep the survey short, avoid dropout when entering symptoms for a longer period, and avoid introducing any bias because of different algorithmic pathways resulting from participants unreliably entering information, which is a nonneglible risk, as shown by Jungmann et al [66]. As trust could be influenced by user experience throughout the interaction [45], we could not account for a potentially moderating role of that factor. This limitation applies equally to all experimental groups; thus, internal validity is not compromised. However, as symptom checkers commonly require extensive user interaction over a span of multiple minutes [67], their ecological validity might be limited. Future research should alter the existing symptom checkers to test whether our results can be replicated in practice. Our participants also only evaluated a single case vignette, whereas, in other studies, participants solved as many as 20 with the help of a symptom checker app. Hence, the duration of exposure to the intervention was low in our study. However, we consider this closer to the real use case of symptom checkers, where users seek advice on a single set of complaints rather than systematically testing the app by iteratively entering the signs and symptoms of highly heterogeneous fictitious patient descriptions. However, unlike in the real use case, participants could not change their decision at a later stage. In practice, they might decide to see a health care professional after gathering further evidence, even if they decided for self-care to be sufficient when using a symptom checker. Thus, our concept of behavioral trust only captures users’ intentions after consulting a symptom checker, not their actual behavior (ie, [not] seeking health care according to the symptom checker’s prompt).

All participants appraised only a single case vignette, which was the same across all 3 groups. We used only this specific case vignette as it has been used in previous studies and was ambiguous enough for patients to choose both self-care and health care. However, technically, many other vignettes and symptoms can be entered and should thus be investigated in the future. The gold standard for the case vignette used in this study is self-care; however, visiting a health care professional with these symptoms is not inappropriate and, in particular, not unsafe. Thus, deviation from the gold standard solution may be considered wrong but not consequential. Although the gold standard solution was assigned by a panel of physicians, the idea of absolute correct urgency may vary for different physicians. It would be interesting to see whether our findings can be replicated for a variety of cases with different gold standard urgency levels (eg, 3-tiered or 4-tiered urgency levels). Other decisions, such as whether emergency care is required, should also be examined, as this study could not provide any evidence for other urgency-related decisions. Especially concerning the decision of whether emergency care is required, we consider a further investigation into the question of whether layperson trust is unaffected by the direction of the (contradicting) advice by a decision aid worthwhile, as here, an incorrect appraisal is more consequential.

It cannot be ruled out that some participants researched the correct solution on the web to obtain a bonus. However, as this could have occurred in all groups, internal validity should not be impaired.

Participants did not assess their own symptoms but were presented with a fictitious case vignette as a proxy for a medical case. Although this arguably reductionist approach is commonly applied when evaluating symptom checkers [6,8,68,69], it remains unclear whether participants assess these symptoms in the same way they do when experiencing them. For example, in the case of real symptoms, not only might the information input change, but the patients’ mental well-being and their perceived self-efficacy in implementing an action might also have an impact. It is also conceivable that participants might not have empathized enough with the situation or that the urgency was assessed differently. However, web-based health information sources are commonly used to assess the symptoms of others [3]; thus, this use case still possesses a high degree of external validity.

As we only collected quantitative data, we could not explain why the participants changed their decisions. Future studies should conduct qualitative studies on decision-making when assisted by a symptom checker.

Finally, the participants in this study were well-educated, with 54.5% (269/494) of participants having a bachelor’s degree or higher. Although our sample is not representative of the US population, the average education level is very close to that of symptom checker users [15]. The same applies to our participants’ average age, which is very close to that of users [16] and had no impact on our exploratory analyses.

### Practical Implications

Although some developers frame their symptom checkers as anthropomorphic or as an AI, there appears to be no meaningful impact on users’ trust based on our study. Although previous studies found an influence of anthropomorphism on trust in general automation [23-25], we could not extend these findings to symptom checkers. As we kept this study as true to reality as possible—by specifically using a mock symptom checker instead of other decision aids used in experimental laboratory setups and by testing an externally valid use case where users only assessed a single case vignette and could not estimate symptom checker accuracy—our results are more applicable to the specific use case of symptom checkers. The effect found by other authors presumably materializes only when users can assess a system’s accuracy. As multiple assessments in a row do not correspond to the natural use of symptom checkers, framing (as currently applied) does not seem to provide any benefit in terms of trust.

Although sociodemographic factors appear to have an impact on symptom checker use [15,16], they do not seem to alter trust. Thus, trust depends on eHealth literacy; for example, symptom checkers do not need to be customized for age, gender, or education to increase trust, although they might be customized to increase usability and user experience.

Finally, as initial trust is very high, regardless of framing and demographic factors, further increasing users’ trust in these systems may not be a priority. Instead, we suggest that it may be more worthwhile to explore ways of supporting users in their decision-making so that they do not have to rely uncritically on a symptom checker’s advice. For example, this can be achieved by providing explanations of disposition advice tailored to the individual user [60].

### Conclusions

The subjective and behavioral trust of laypersons in clinical decision aids is high and is not influenced by framing such systems as anthropomorphic or using AI.

However, users are inclined to change their minds based on the symptom checker’s advice, even when they report maximum certainty in their initial and contradicting stand-alone appraisal. This indicates the high persuasive power of the symptom checker and thus demonstrates its potential to make patient journeys more efficient. At the same time, our findings hint at the danger that laypersons may use symptom checkers to substitute rather than to assist their own decision-making. Although some symptom checkers commonly provide accurate and safe advice, the range of symptom checker accuracy varies widely. Thus, before recommending symptom checkers for general use, rigorous standards for evaluating symptom checkers must be defined to ensure that only those symptom checkers are recommended that are accurate and safe enough to be worthy of the trust people have in them. Further research should investigate how to ensure that symptom checkers function as aids rather than replacements in laypersons’ decision-making.

## Appendix

Multimedia Appendix 1 Effect coding scheme of gender.

Multimedia Appendix 2 Effect coding scheme of education.

Multimedia Appendix 3 Multiple linear regression of demographic and interindividual influences on subjective trust with standardized coefficients.

Multimedia Appendix 4 Multiple binomial logistic regression of demographic and interindividual influences on behavioral trust with standardized coefficients.

Multimedia Appendix 5 Multiple linear regression of demographic and interindividual influences on subjective trust with unstandardized coefficients.

Multimedia Appendix 6 Multiple binomial logistic regression of demographic and interindividual influences on behavioral trust with unstandardized coefficients.

Multimedia Appendix 7 CONSORT-eHEALTH checklist (V 1.6.1).

## Abbreviations

AI: artificial intelligence
EFS: Enterprise Feedback Suite
OR: odds ratio
