# Supplementary material for: Determinants of Laypersons’ Trust in Medical Decision Aids: Randomized Controlled Trial
Source: JMIR Hum Factors. 2022 May 3;9(2):e35219. doi: 10.2196/35219 (PMC9115664; doi:10.2196/35219)
Supplement: Multimedia Appendix 1 [file humanfactors_v9i2e35219_app1.docx]

Multimedia Appendix 1. Effect coding scheme of gender.

| Factor Level | Gender 1 | Gender 2 |
| --- | --- | --- |
| Female | 1 | 0 |
| Other | 0 | 1 |
| Male | -1 | -1 |
