# Supplementary material for: Determinants of Laypersons’ Trust in Medical Decision Aids: Randomized Controlled Trial
Source: JMIR Hum Factors. 2022 May 3;9(2):e35219. doi: 10.2196/35219 (PMC9115664; doi:10.2196/35219)
Supplement: Multimedia Appendix 2 [file humanfactors_v9i2e35219_app2.docx]

Multimedia Appendix 2. Effect coding scheme of education.

| Factor Level | Education 1 | Education 2 | Education 3 | Education 4 |
| --- | --- | --- | --- | --- |
| Bachelor Degree | 1 | 0 | 0 | 0 |
| College or Associate Degree | 0 | 1 | 0 | 0 |
| Graduate Degree & Higher | 0 | 0 | 1 | 0 |
| High School Graduate | 0 | 0 | 0 | 1 |
| Less Than High School | -1 | -1 | -1 | -1 |
