# Supplementary material for: Determinants of Laypersons’ Trust in Medical Decision Aids: Randomized Controlled Trial
Source: JMIR Hum Factors. 2022 May 3;9(2):e35219. doi: 10.2196/35219 (PMC9115664; doi:10.2196/35219)
Supplement: Multimedia Appendix 3 [file humanfactors_v9i2e35219_app3.docx]

Multimedia Appendix 3. Multiple linear regression of demographic and interindividual influences on subjective trust with standardized coefficients.

| Predictor | b | SE |  | P |
| --- | --- | --- | --- | --- |
| Intercept | -0.049 | 0.186 | -0.263 | .793 |
| Age | 0.065 | 0.044 | 1.480 | .139 |
| Gender 1 | -0.045 | 0.115 | -0.392 | .695 |
| Gender 2 | -0.018 | 0.213 | -0.084 | .933 |
| Education 1 | 0.022 | 0.098 | 0.228 | .820 |
| Education 2 | 0.190 | 0.099 | 1.909 | .057 |
| Education 3 | 0.008 | 0.117 | 0.066 | .948 |
| Education 4 | 0.106 | 0.126 | 0.839 | .402 |
| Basic First Aid Training (Yes) | -0.034 | 0.115 | -0.300 | .764 |
| Propensity to Trust | 0.249 | 0.044 | 5.616 | <.001 |
| eHealth Literacy | 0.200 | 0.044 | 4.566 | <.001 |

Beta Coefficient. SE = Standard Error. t = t-Value. P = p-Value. Total variance explained is R^2^_Adjusted_ = .117. The effect coding scheme for Gender and Education can be found in the Multimedia Appendices 1 and 2. For example, Gender 1 represents the comparison of females with the mean of the other genders. Education 1 represents the comparison of participants with a Bachelor degree with the mean of all other education levels.
