# Supplementary material for: Determinants of Laypersons’ Trust in Medical Decision Aids: Randomized Controlled Trial
Source: JMIR Hum Factors. 2022 May 3;9(2):e35219. doi: 10.2196/35219 (PMC9115664; doi:10.2196/35219)
Supplement: Multimedia Appendix 4 [file humanfactors_v9i2e35219_app4.docx]

Multimedia Appendix 4. Multiple binomial logistic regression of demographic and interindividual influences on behavioral trust with standardized coefficients.

| Predictor | b | SE | t | P |
| --- | --- | --- | --- | --- |
| Intercept | -0.900 | 0.530 | 1.700 | .089 |
| Age | -0.104 | 0.110 | -0.940 | .347 |
| Gender 1 | -0.363 | 0.376 | -0.965 | .335 |
| Gender 2 | 0.486 | 0.717 | 0.678 | .498 |
| Education 1 | 0.391 | 0.234 | 1.673 | .094 |
| Education 2 | 0.442 | 0.239 | 1.850 | .064 |
| Education 3 | 0.055 | 0.272 | 0.204 | .839 |
| Education 4 | -0.105 | 0.293 | -0.360 | .719 |
| Basic First Aid Training (Yes) | 0.281 | 0.313 | 0.896 | .370 |
| Propensity to Trust | -0.045 | 0.114 | -0.400 | .689 |
| eHealth Literacy | 0.004 | 0.114 | 0.032 | .975 |

b = Beta Coefficient. SE = Standard Error. z = z-Value. P = p-Value. Total variance explained is McFaddens R^2^ = .007. The effect coding scheme for Gender and Education can be found in the Multimedia Appendices 1 and 2. For example, Gender 1 represents the comparison of females with the mean of the other genders. Education 1 represents the comparison of participants with a Bachelor degree with the mean of all other education levels.
