# Supplementary material for: Determinants of Laypersons’ Trust in Medical Decision Aids: Randomized Controlled Trial
Source: JMIR Hum Factors. 2022 May 3;9(2):e35219. doi: 10.2196/35219 (PMC9115664; doi:10.2196/35219)
Supplement: Multimedia Appendix 5 [file humanfactors_v9i2e35219_app5.docx]

Multimedia Appendix 5. Multiple linear regression of demographic and interindividual influences on trust with unstandardized coefficients.

| Predictor | b | SE | t | P |
| --- | --- | --- | --- | --- |
| Intercept | 1.467 | 0.405 | 3.625 | <.001 |
| Age | 0.004 | 0.03 | 1.480 | .139 |
| Gender 1 | -0.040 | 0.102 | -0.392 | .695 |
| Gender 2 | -0.016 | 0.189 | -0.084 | .933 |
| Education 1 | 0.020 | 0.087 | 0.228 | .820 |
| Education 2 | 0.168 | 0.088 | 1.909 | .057 |
| Education 3 | 0.007 | 0.104 | 0.066 | .948 |
| Education 4 | 0.094 | 0.112 | 0.839 | .402 |
| Basic First Aid Training (Yes) | -0.031 | 0.102 | -0.300 | .764 |
| Propensity to Trust^1^ | 0.447 | 0.080 | 5.616 | <.001 |
| eHealth Literacy^2^ | 0.034 | 0.007 | 4.566 | <.001 |

^1^ Possible Values: 1-5

^2^ Possible Values: 8-40
