# Supplementary material for: Determinants of Laypersons’ Trust in Medical Decision Aids: Randomized Controlled Trial
Source: JMIR Hum Factors. 2022 May 3;9(2):e35219. doi: 10.2196/35219 (PMC9115664; doi:10.2196/35219)
Supplement: Multimedia Appendix 6 [file humanfactors_v9i2e35219_app6.docx]

Multimedia Appendix 6. Multiple binomial logistic regression of demographic and interindividual influences on follow rates with unstandardized coefficients.

| Predictor | b | SE | t | p |
| --- | --- | --- | --- | --- |
| Intercept | 1.518 | 1.196 | 1.269 | .204 |
| Age | -0.008 | 0.009 | -0.940 | .347 |
| Gender 1 | -0.363 | 0.376 | -0.965 | .335 |
| Gender 2 | 0.486 | 0.717 | 0.678 | .498 |
| Education 1 | 0.391 | 0.234 | 1.673 | .094 |
| Education 2 | 0.442 | 0.239 | 1.850 | .064 |
| Education 3 | 0.055 | 0.272 | 0.204 | .839 |
| Education 4 | -0.105 | 0.293 | -0.360 | .719 |
| Basic First Aid Training (Yes) | 0.281 | 0.313 | 0.896 | .370 |
| Propensity to Trust^1^ | -0.092 | 0.230 | -0.400 | .689 |
| eHealth Literacy^2^ | 0.001 | 0.022 | 0.032 | .975 |

^1^ Possible Values: 1-5

^2^ Possible Values: 8-40
